# Community-based social determinants of three measures of mortality in Rhode Island cities and towns

**DOI:** 10.1186/s13690-020-00438-7

**Published:** 2020-06-15

**Authors:** Steven A. Cohen, Julia R. Broccoli, Mary L. Greaney

**Affiliations:** Department of Health Studies, University of Rhode Island, 25 West Independence Way Suite P, Kingston, Rhode Island 02881 USA

**Keywords:** Social determinants of health, Small-area measures, Geographic analysis, Demographic methods

## Abstract

**Background:**

Efforts to understand and address the causes of place-based health disparities have focused primarily on understanding the social determinants of health on a large geographic level, such as the region, state, or county. However, there is a growing need to assess and understand how place-based characteristics at smaller geographic areas relate to of local place-based neighborhood characteristics on population health. Therefore, the objective of this study was to evaluate the magnitude of the associations between social determinants of health and life expectancy (LE) and related measures on the community level.

**Methods:**

LE at birth (LE0), remaining LE at age 65 (LE65), and age-specific mortality rates (ASMR) were calculated from mortality data (2009–2011) collected by the Rhode Island Department of Health (RIDoH) using abridged life table methods for each RI city/town. The city/town-specific LE and ASMR were linked to data collected by the US Census, RIDoH, the Federal Bureau of Investigation, and other databases that include information about multiple social, environmental, and demographic determinants of health. Bivariate correlations between city/town-level LE0, LE65, and ASMR and social determinants: demographics, household composition, income and poverty, education, environment, food insecurity, crime, transportation, and rural-urban status were examined.

**Results:**

LE0 (range: 75.9–83.3 years) was strongly associated with the percent of the population with a graduate/professional degree (*r* = 0.687, *p* <  0.001), violent crime rate (*r =* − 0.598, *p <*  0.001), and per capita income (*r =* 0.553, *p* <  0.001). Similar results were observed for ASMR: ASMR was associated with the percent of the population with a graduate/professional degree (*r =* − 0.596, *p <*  0.001), violent crime rate (*r =* 0.450, *p* = 0.005), and per capita income (*r =* − 0.533, *p* < 0.001). The associations between LE65 and social determinants were more attenuated. Of note, none of the measures (LE0, LE65, or ASMR) were associated with any of the race/ethnicity variables.

**Conclusions:**

There are several important place-based characteristics associated with mortality (LE and ASMR) among RI cities/towns. Additionally, some communities had unexpectedly high LE and low ASMR, despite poor social indicators.

## Background

Place matters for population health. Evidence suggests that one’s place of residence plays a substantial and important role in determining individual health status in the United States and many other nations [1]. As a result, health inequities based on geography occur [2–5]. A number of studies have demonstrated health disparities by geography, examples include, but are not limited to, cancer [6], physical activity and obesity [7], health care quality and access [8–10], and cancer screening [11, 12].

Research to understand and address the causes of place-based health disparities has focused primarily on social determinants of health within a large geographic level, such as a region, state, or county [13]. Recently, however, there has been growing interest in drilling down to the local level and assessing health disparities at smaller geographic areas to evaluate the influence of place-based neighborhood and municipality characteristics [14]. The reason for this is that policies, demographic characteristics, and economic conditions at the local level potentially affect availability and quality of resources, community development, and economic opportunities [15]. Increasingly, research suggests that understanding how social determinants, including education, wealth, crime, environmental factors, housing, and numerous others at a smaller geographic area influence population health is critical to ameliorating health inequities occurring within these small geographic areas [16–23].

Life expectancy is a widely used summary measure of population health, and represents the average lifespan based on current death rates, and provides a global picture of population health [24]. In the United States, differences in LE by place are substantial and have increased over time [25]. Consider, for example, the US county with the lowest LE (Oglala Lakota County in South Dakota, 66.8 years) and the county with the highest LE (Summit County in Colorado, 86.8 years), a difference of 20 years. That stark difference in LE between these two counties located only 400 miles from one another is nearly equivalent to the difference in LE between women in Ethiopia (66.7 years) and Japan (86.8).

There is a growing body of research highlighting the utility of employing LE to document health inequities, and to determine and understand what health disparities also occur at fine geographic levels that are smaller units than county, state, or nation. These include the city or town (municipality), census tract, and neighborhood levels [26–30]. Differences at this more granular level may be masked when LE or other health-related metrics are calculated on a larger level, such as the county or state level [31, 32], geographic levels on which most spatial research has been conducted. Nonetheless, no studies to date have quantified LE at a fine geographic level (e.g. sub-county) for a large geographic area and systematically assessed potential associations between LE and social determinants within that fine geographic level. To that end, the aims of this exploratory study were to: (1.) estimate LE and related measures of population health in all Rhode Island (RI) municipalities; (2.) compare and contrast life expectancy LE at birth with other population health measures; and (3.) explore associations between key measures of population health (LE and mortality) and an array of social determinants.

## Methods

### Outcome measures: life expectancy and age-standardized mortality rates

LE for this study was calculated using methods adapted from the CDC’s methodology for calculating LE on a small geographic scale [33]. All de-identified death records for 2009, 2010, and 2011 from the RI Department of Health Center for Vital Records were geocoded to each of the 39 RI cities and towns (“municipality”). All deaths during this same time period were summed to create tables of total deaths in each RI municipality by 5-year age groups. These data were then paired with detailed population counts for each of the 5-year age groups for all RI municipalities and then used to obtain age-specific death rates using population data from the 2010 US Decennial Census. From this information, LE at birth (LE0) and at age 65 (LE65) were calculated with associated standard errors and 95% confidence intervals for each municipality. In total, 13% of the 663 cells had zero deaths. In these cases, the RI age-specific death rate was used to calculate LE. As a result, the calculated LEs for these municipalities may be slightly biased toward the mean LE [34, 35]. Age-standardized mortality rates (ASMRs) for each RI municipality also were derived using the calculated age-specific death rates with the same substitution method applied for cells with zero death counts.

### Exposure measures: social determinants of health

A set of 55 social determinants of health and related measures was obtained from the most recent (2010) US Decennial Census and American Community Survey and other sources (e.g. RI Kids Count, Youth Risk Behavior Surveillance System, the Federal Bureau of Investigation) to create a database of 89 social determinants that included measures of education, income and wealth, poverty, green space, crime, demographics, housing, household composition, rural/urban characteristics, environment, food insecurity, land use, transportation, commuting, and age distribution. A detailed list of all examined determinants is provided in the Results section.

### Data analysis

Descriptive statistics were obtained for the three main summary measures of population health—LE at birth (LE0), LE at age 65, (LE65), and ASMR—and for each of the social determinants for each RI municipality, including means, standard deviations, minima, and maxima for all continuous and discrete variables, and frequency distributions for all categorical variables. Shapefiles for all municipalities were obtained and downloaded for use in mapping from the RI Geographic Information System [36]. Using geographic information systems (GIS) software, detailed choropleth maps of LE0, LE65, ASMR, and the examined social determinants for all RI municipalities were created.

Pearson and Spearman correlations were used to estimate the bivariate associations between the three outcome measures (LE0, LE65, and ASMR) and the examined social determinants. Rank order variables (e.g. school ranking, etc.) had identical Pearson and Spearman correlation values. Linear regression models were constructed to estimate associations and determine which factors are predictive of LE0, LE65, and ASMR using forward stepwise methods. Model assumptions for linear regression were checked for the statistical “best” model for each of the outcome measures. Resultant associations between the health outcomes (LE0, LE65, and ASMR) and the social determinants were then examined using meta-regression [37], employing permutation tests and bootstrapping to adjust the *p*-values for multiplicity [38]. Although there is possible geo-spatial autocorrelation, for purposes of modeling, each municipality was considered to be independent of the other municipalities [39]. IBM SPSS version 26 (Armonk, NY) and SAS version 9.4 (Cary, NC) were used for all data management and analysis. ArcGIS version 10.1 (Redlands, WA) was used for all mapping and geospatial analysis. SPSS and Microsoft Excel were used for graphing. Statistical significance was set at alpha < 0.05. This study was approved by the University of Rhode Island Institutional Review Board (protocol #1259971–2).

## Results

Information on LE0, LE65, and ASMR for all RI municipalities can be found in Table 1. The average LE0 in RI was 79.92 years, with a standard deviation of 1.60. There was a 7.3-year difference between the town with the highest LE0 (Barrington, 83.13 years) and the lowest LE0 (Woonsocket, 75.85 years). Barrington also had the lowest ASMR (722.0 per 100,000), while the highest ASMR (1127.6 per 100,000) was found in Woonsocket. New Shoreham had the highest LE65 (21.92 years), while Richmond had the lowest LE65 (17.59 years).

**Table 1:** Rhode Island cities/towns, life expectancy (LE), and age-standardized mortality rates (ASMR)

**City/Town Name**	**County**	**LE at birth (LE0)**	**LE at age 65 (LE65)**	ASMR*	**Rank LE at birth**	**Rank LE 65**	**Rank of ASMR**
Barrington	Bristol	83.13	21.34	722.01	1	3	1
Bristol	Bristol	80.94	20.18	852.95	12	15	15
Burrillville	Providence	77.33	18.22	1052.02	37	37	37
Central Falls	Providence	78.26	20.06	970.74	35	19	33
Charlestown	Washington	80.75	20.41	844.02	14	14	13
Coventry	Kent	79.31	18.73	963.18	27	35	32
Cranston	Providence	81.32	20.64	819.72	8	10	9
Cumberland	Providence	81.50	20.79	799.52	5	8	4
East Greenwich	Kent	82.17	20.83	782.78	3	7	3
East Providence	Providence	79.61	19.72	920.91	22	25	25
Exeter	Washington	79.59	20.17	897.68	23	16	20
Foster	Providence	79.14	21.16	813.75	28	4	8
Glocester	Providence	80.09	20.02	848.50	18	22	14
Hopkinton	Washington	78.90	19.70	916.36	30	26	23
Jamestown	Newport	80.83	20.89	893.34	13	6	17
Johnston	Providence	79.40	19.53	939.39	25	30	28
Lincoln	Providence	81.26	20.15	835.33	10	17	12
Little Compton	Newport	81.32	21.02	812.48	8	5	7
Middletown	Newport	80.26	19.66	893.38	17	28	18
Narragansett	Washington	82.00	20.52	799.92	4	13	5
New Shoreham	Washington	76.76	21.92	897.65	38	1	19
Newport	Newport	79.52	19.97	917.56	24	23	24
North Kingstown	Washington	80.34	19.45	945.22	15	31	29
North Providence	Providence	79.91	20.58	872.42	20	12	16
North Smithfield	Providence	79.40	19.67	927.55	25	27	27
Pawtucket	Providence	78.94	19.75	946.90	29	24	30
Portsmouth	Newport	82.93	21.46	727.48	2	2	2
Providence	Providence	78.37	19.38	973.77	34	32	34
Richmond	Washington	78.43	17.59	1072.26	33	39	38
Scituate	Providence	81.43	20.64	807.14	6	10	6
Smithfield	Providence	80.34	19.25	923.54	15	34	26
South Kingstown	Washington	81.34	20.07	833.63	7	18	11
Tiverton	Newport	81.16	20.72	830.02	11	9	10
Warren	Bristol	79.94	20.05	906.42	19	21	22
Warwick	Kent	78.89	19.31	959.59	31	33	31
West Greenwich	Kent	78.65	18.44	1029.60	32	36	36
West Warwick	Kent	77.79	19.56	985.23	36	29	35
Westerly	Washington	79.64	20.06	901.35	21	19	21
Woonsocket	Providence	75.85	18.19	1127.58	39	38	39

Note: *ASMR* Age-standardized mortality rate

*Per 100,000

Descriptive statistics for the major population health measures and examined social determinants can be found in Table 2. There was a wide range of values for several demographic variables, including population density, which ranged from 91 people per square mile in Foster to 16,172 people per square mile in Central Falls. Likewise, the percent of population that is Hispanic/Latino/a ranged from 0.3% in Glocester to 87.4% in Central Falls. Descriptive statistics for crime, economic, education, environmental and recreational, and retirement-based measures are also provided in Table 2. The economic variable “median home value”, cannot exceed the maximum of $1000,000 as measured by the US Census, found in New Shoreham, so the actual value in New Shoreham may be higher. The percent of family households headed by females alone ranged from 3.4% in New Shoreham to 21.7% in Providence. Maps for key population health measures and social determinants are located in the **Supplementary Material**).

**Table 2:** Descriptive statistics for all variables examined

**Measure**	**Mean**	**SD**	**Min**	**Max**	**Number missing**
**Population Health**					
Life expectancy at birth (LE0)	79.92	1.60	75.85	83.13	0
Remaining life expectancy at age 65 (LE65)	19.99	0.93	17.59	21.92	0
Age-standardized mortality rate (ASMR)	896.5	89.2	722.0	1127.6	0
**Demographics**					
% White	90.9	10.0	49.1	99.5	0
% Black	3.0	4.1	0.0	17.5	0
% American Indian/Native Hawaiian	0.3	0.4	0.0	1.6	0
% Asian	1.7	1.6	0.0	6.4	0
% Other race	2.2	4.7	0.0	24.3	0
% Multiracial	1.8	1.1	0.0	5.0	0
% Hispanic/Latino/a	8.3	18.2	0.3	87.4	0
Black-White Index of Dissimilarity	41.8	15.9	9.7	72.2	5
% of population aged 16+	81.7	2.9	74.6	87.0	0
% of population aged 65+	15.5	3.4	8.7	22.7	0
% foreign born	7.7	7.9	1.6	41.8	0
Population density (people per square mile)	2085	3199	91	16,172	0
% of population considered “rural	27.6	36.9	0.0	100.0	0
People per housing unit	2.2	0.4	0.6	2.7	0
% of households headed by married couple	52.4	10.4	29.5	72.0	0
% of family households headed by single female	10.4	4.0	3.4	21.7	0
**Education**					
% of adults with less than 9th grade education	4.9	5.5	0.0	30.8	0
% of adults with less than high school education	12.6	8.6	2.3	48.2	0
% of adults with at least a high school education	87.4	8.6	51.7	97.7	0
% of adults with at least a bachelor’s degree	34.3	12.8	7.4	63.4	0
% of adults with a graduate degree	13.5	6.6	2.0	30.7	0
**Economics**					
Median household income ($)	85,605	21,406	40,526	140,772	0
Median home value ($)	310,062	140,488	167,600	1,000,000	0
Median rent ($)	965	178	731	1403	0
Poverty rate	5.9	5.4	0.6	24.6	0
% on public assistance	7.1	6.8	0.0	30.7	0
% unemployed	0.1	0.0	0.0	0.1	0
Gini index	0.43	0.04	0.34	0.53	0
Mean commute time (minutes)	24.2	4.4	8.9	35.7	0
**Crime rates (per 1000)**					
All violent crimes	1.45	1.76	0.00	7.33	2
Murder and non-negligent manslaughter	0.01	0.02	0.00	0.10	2
Forcible rape	0.16	0.18	0.00	0.72	2
Robbery	0.32	0.56	0.00	2.43	2
Aggravated assault	0.95	1.07	0.00	4.13	2
All property crimes	19.75	9.20	4.31	44.84	2
Burglary	4.06	2.46	1.23	10.84	2
Larceny	14.42	6.64	2.46	33.46	2
Motor vehicle theft	1.28	1.55	0.00	6.54	2
Arson	0.14	0.14	0.00	0.49	2
**Environmental and recreational factors**					
Number of Superfund sites	0.3	0.6	0	2	0
Median age of housing structures	48.3	12.2	24.0	74.0	0
% of households without plumbing	1.2	0.9	0.0	3.6	0
% of land area used for public recreation	16.9	8.8	4.3	42.6	0
Miles of bike lanes per 50 road miles	1.5	1.4	0.0	4.5	0
% living near farmers market	50.2	32.9	0.0	99.6	0
Fast food and convenience stores per square mile	4.3	7.4	0.1	37.2	0
% of population living in food desert	31.9	28.0	0.0	100.0	0
% of total area comprised of water	20.8	23.8	1.1	91.7	0
**Retirement**					
% of grandparental caregivers who are male	36.6	11.3	0.0	59.3	1
% of grandparents responsible for grandchildren	29.8	20.8	0.0	78.0	1
% of grandparents living in poverty	7.7	17.1	0.0	100.0	1
Mean per capita retirement income ($)	24,985	6387	16,111	42,238	0

**Fig. 1:**
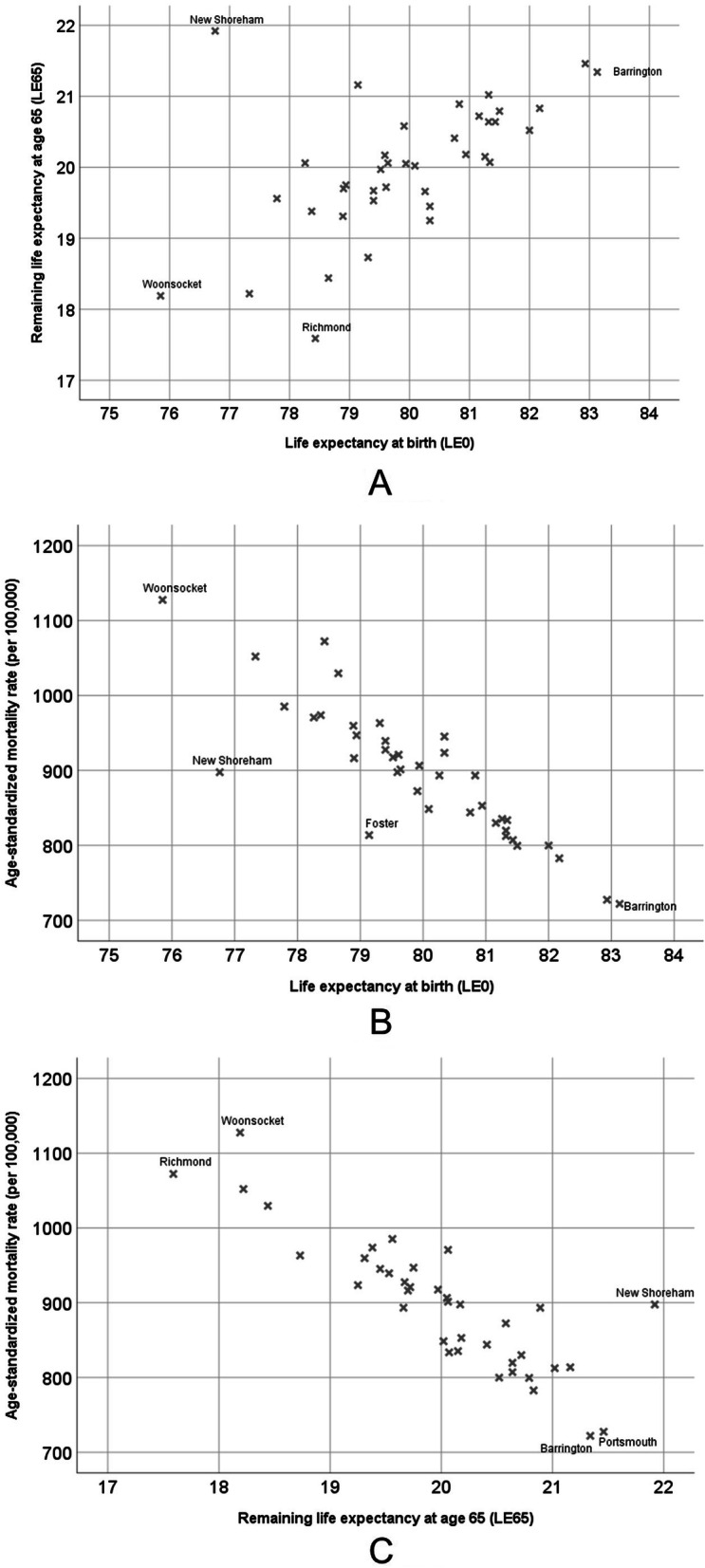
Associations between LE0 and LE65 (Panel **a**), LE0 and ASMR (Panel **b**) and LE65 and ASMR (Panel **c**)

The bivariate associations among the three primary measures of population health (LE0, LE65, and ASMR) were moderate to strong; the Pearson correlation between LE0 and LE65 was 0.578 (*p* < 0.001). There were several outliers of note (see Fig. 1**, Panel A**), including New Shoreham and Richmond. Although New Shoreham has the second-lowest LE0 in RI (76.8 years), it had the highest LE65 (21.9 years). Richmond had the lowest LE65 (17.6 years), and was ranked 33rd highest out of all 39 RI municipalities for LE0 (78.4 years). The Pearson correlation between LE0 and ASMR was − 0.872 (*p* < 0.001), and between LE65 and ASMR was − 0.863 (*p <* 0.001) (Fig. 1**, Panels B and C**).

**Fig. 2:**
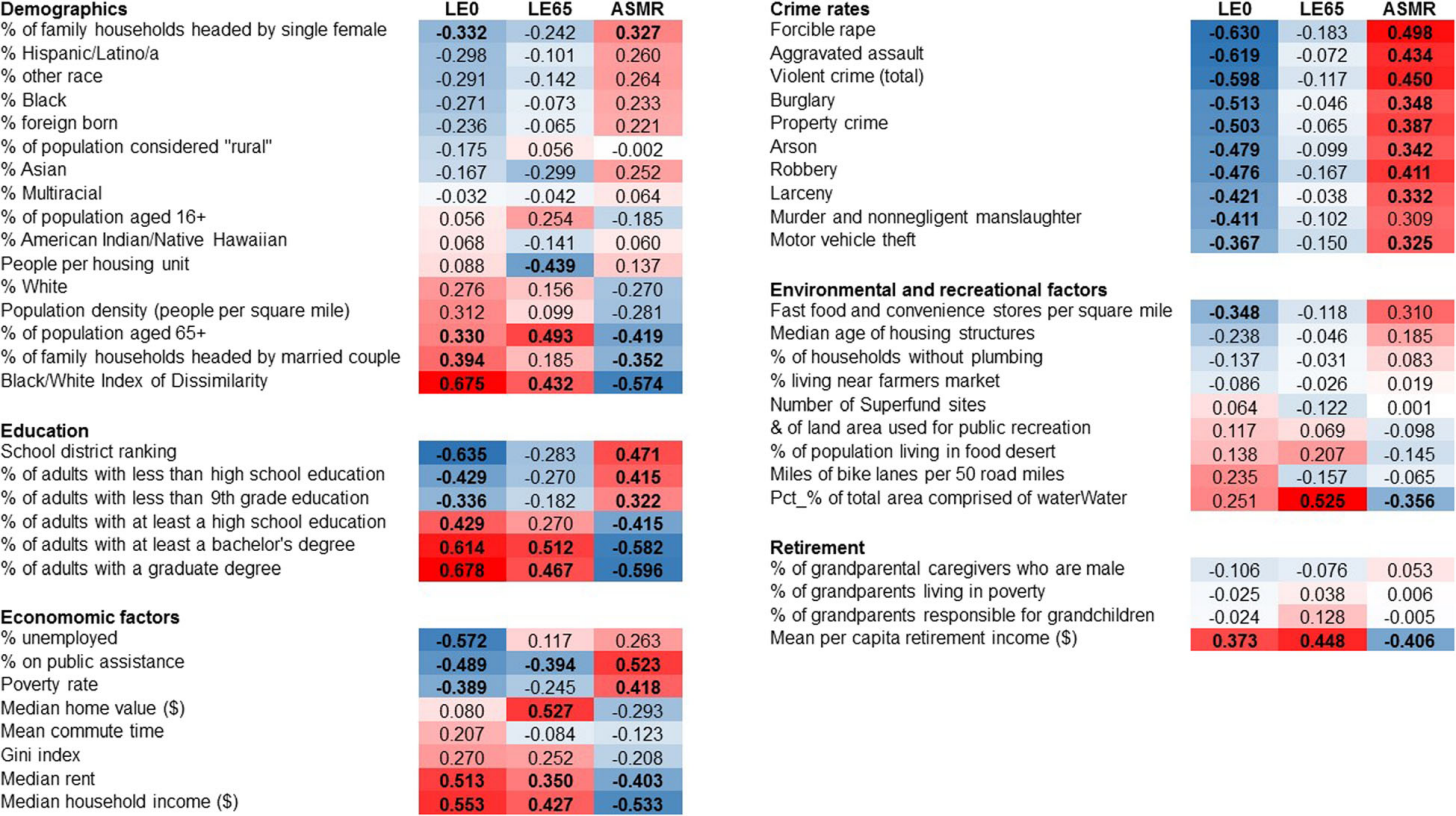
Pearson correlation between LE0, LE65, and ASMR and each of the social determinants examined. The magnitude and direction of the correlation is shown by color: red and pink indicate positive correlations, while blue indicates negative correlations. The darker the color, the stronger the correlation. Boldface = significant at *p* < 0.05

Correlations between LE0, LE65, and ASMR and each of the exposure measures are shown in Fig. 2. In this figure, the measures are categorical, and ranked within category by magnitude and direction of correlation (smallest to largest) between the measure and LE0. For the demographic variables, the percent of family households headed by a single female was negatively associated with LE0 (*r =* − 0.332, *p* = 0.039), and positively correlated with the percent of population aged 65+ (*r =* 0.330, *p* = 0.040), the percent of family households headed by a married couple (*r =* 0.394, *p* = 0.013), and the Black/White Index of Dissimilarity, a measure of segregation (*r =* 0.675, *p* < 0.001). For example, a city or town with an index of dissimilarity of 0.55 indicates that 55% of White people would need to move to another census tract within that city or town to distribute Whites and Blacks evenly across all census tracts in that city or town. Similar results, but in the opposite direction, were found for these measures and ASMR. People per housing unit was negatively correlated with LE65 (*r =* − 0.439, *p* = 0.005). LE0 and ASMR were significantly associated with the six examined education variables, but the associations between LE65 and ASMR were only significant for the percent of adults with at least a bachelor’s degree (*r =* 0.512, *p* = 0.001) and with a graduate degree (*r =* 0.467, *p* = 0.003).

The results of this analysis examining economic factors were more varied. Percent unemployed was strongly and negatively associated with LE0 (*r =* − 0.572, *p* < 0.001), but was not significantly associated with either LE65 or ASMR. Percent of population on public assistance, median rent, and median household income were all significantly associated with LE0, LE65, and ASMR. Poverty rate was associated with LE0 (*r =* − 0.389, *p* = 0.014) and ASMR (*r =* 0.418, *p* = 0.008), but not with LE65 (*r =* − 0.245, *p* = 0.113). LE65, however, was significantly associated with median home value (*r =* 0.527, *p* = 0.001) while LE0 and ASMR were not. LE0 and ASMR were significantly associated with all of the seven examined individual crime rate measures, as well as overall violent crime and property crime rates, while LE65 was not significantly associated with any of these measures.

Fast food and convenience stores per square mile was significantly associated with decreased LE0 (*r =* − 0.348, *p* = 0.030), while the percent of total area of the city or town comprised of water (lakes, ponds, bay, ocean, etc.) was significantly associated with both LE65 (*r =* 0.525, *p* = 0.001) and ASMR (*r =* − 0.356, *p* = 0.026), but not with LE0 (*r =* − 0.251, *p* = 0.122). Mean per capita retirement income was the only retirement measure that was significantly associated with LE0, LE65, and ASMR.

For LE0, three social determinants remained significant in the forward stepwise linear regression model (Table 3): increasing percentages of adults with at least a bachelor’s degree (beta = 0.11, *p* < 0.001), percentage of the population aged 65+ (beta = 0.23, *p* = 0.001), and percentage of multigenerational households (beta = 0.43, *p* = 0.024) were all significant predictors of LE0, and remained so in the meta-regression models. Only median rent (beta = 0.003, *p* = 0.001) remained significantly associated with LE65, but was not significant in the meta-regression model. Median rent also was significantly associated with ASMR, and with five other social determinants: percentage of adults with a graduate degree, percentage of population aged 65+, average commuting time (minutes), percentage foreign born, and percentage on public assistance, although percent 65+, commuting time, and median rent were not significant in the meta-regression models. The model parameters explained 70.9, 28.7, and 78.2% of the variability in LE0, LE65, and ASMR, respectively.

**Table 3:** Model parameter estimates (and 95% confidence intervals) and model fit statistics for three population health outcomes from forward stepwise regression

	**Beta (95% CI)**	*P*-value*	*P*-value**	**R**^**2**^	**Adjusted R**^**2**^
**LE0**				0.709	0.680
% of adults with at least a bachelor’s degree	0.11 (0.08, 0.15)	< 0.001	0.001		
% of population aged 65+	0.23 (0.10, 0.35)	0.001	0.018		
% multigenerational households	0.43 (0.06, 0.80)	0.024	0.039		
Constant	71.63 (68.53, 74.72)				
**LE65**				0.287	0.265
Median rent ($)	0.003 (0.001, 0.005)	0.001	0.073		
Constant	16.92 (15.19, 18.64)				
**ASMR**				0.782	0.734
% of adults with a graduate degree	−5.92 (−10.62, −1.22)	0.015	0.020		
% of population aged 65+	−12.39 (−21.21, −3.57)	0.008	0.107		
Commuting time (minutes)	−10.39 (−16.09, −4.70)	0.001	0.068		
% foreign born	−8.51 (−12.35, −4.67)	< 0.001	0.002		
% on public assistance	7.72 (2.16, 13.29)	0.008	0.008		
Median rent	−0.20 (−0.38, −0.01)	0.043	0.734		
Constant	1635.44 (1308.57, 1962.31)				

Note: LE = life expectancy

*From linear regression

**From meta-regression using linear modeling

## Discussion

Education and crime were consistent correlates of both LE0 and ASMR, although several other social determinants were associated with these measures. Social determinants, in general, explained a substantial portion of the variability in LE0 and ASMR, but explained substantially less variability in LE65. Study findings validate previous research showing that population health is associated with a variety of social determinants, including education, wealth, crime, and household composition. Although the same age-specific mortality rates were used to calculate LE0 and ASMR, there are some discrepancies between the two measures of population health, as there also were differences with respect to the social determinants that were closely correlated with each determinant. These differences could be due to slight differences in how these variables are calculated. LE0 is affected more by variability in mortality rates at younger ages than ASMR, which weights age-specific mortality based on the standard used, in this case, the RI state population. However, correlations between LE0 and ASMR were stronger than correlations between LE0 and LE65 and between ASMR and LE65.

At the national level, the association between education and LE0 is well documented, with several studies finding substantial differences between those with higher education compared to those with less education [25, 40, 41]. Furthermore, one study found that temporal improvements in LE occurred only in more educated population subgroups [42]. The present study’s correlational findings extends prior research by highlighting that the education-LE association exists at a finer geographic level. Many other findings from the present study corroborate prior research on other social determinants, including food insecurity [43], income and wealth [44–46], and crime [47, 48]. Conversely, study findings suggest that, although there were no associations between any of the demographic characteristics (e.g. percent Black, percent Asian, etc.) and LE or ASMR, higher levels of Black-White isolation as measured by the Black-White Index of Dissimilarity, were associated with higher LE0 and lower ASMR. This finding counters the preponderance of evidence suggesting that higher residential segregation worsens population health [49–52]. The differences identified in this study may be due to the estimation of LE and ASMR, which was for the entire population of each RI city or town, irrespective of race/ethnicity. Similar methods have been used in prior studies [53, 54]. Nonetheless, further research is needed to understand the possible reasons for this contradictory finding.

The interpretation of study results should be considered in the context of several important limitations. First, these are cross-sectional data; therefore causality cannot be inferred. Second, study results refer to mortality data from the 2009–2011 timeframe. Patterns of mortality, as well as social determinants, may have changed somewhat between this timeframe and the present. Although more recent mortality data are available from the Rhode Island Department of Health, data from this period were used to correspond closest to the timing of the 2010 US Census data, which was one of the main data sources of social determinants. The 2010 Census is the most recent decennial census, and population data used from the decennial census is more accurate than more recent inter-censal estimates [55]. Third, although 55 social determinants were examined in this correlational analysis, the list of examined social determinants and population health measures (LE0, LE65, and ASMR) are not exhaustive. There are numerous other summary measures of population health, including specific health conditions, healthcare services utilization, and general health indices, that can be assessed, if available, in future studies. Next, spatial autocorrelation is another potential limitation. In this study, each RI municipality was considered to be an independent observation. However, municipalities that are close together may have more in common with each other than those that are further apart. Similarly, study findings are valid only on the geographic level analyzed in this analysis—the municipality level. Findings may be different if analyzed on smaller (e.g. census tract, block group) or larger (e.g. county) scales. Furthermore, small-area LE calculations are subject to substantial error [30, 56, 57]. In the calculation of LE and ASMR, many of the cells (13%) used contained a death count of zero. The state age-specific death rates were used as substitutes for cells with zero death count, which would bias the results toward the mean. Minor changes in the number of deaths, particularly for cities and towns with low death counts in the younger ages, can have a sizeable impact on the calculation of LE and ASMR. Lastly, the small sample size of RI Island municipalities (*n* = 39) limits the overall power of the study, especially with respect to multivariable analyses.

Despite these limitations, this exploratory study has several important strengths. This study is among the first to explore municipal-level social determinants of three measures of population health in a state across an entire state. In RI, as is the case with other northeastern states, local governance is conducted at the municipality (city or town) level. Therefore, study findings can be used at the local governance level to potentially implement policies and programs designed to improve population health and living conditions to reduce geographic disparities in health. Although not all social determinants of health could be obtained for this study, such as literacy, healthcare access, adverse childhood experiences, and others, the list of examined determinants represents a wide breadth of topics and measures, many of which are potentially modifiable. The study results could be used by policymakers, researchers, and the general public, to become informed about RI communities, as well as used as a template for analysis of social determinants of population health in other states and regions, as well. Furthermore, this study, like other studies [6, 8–12, 16–29, 40] also demonstrates that social determinants explain a substantial amount of the variability in population health across geographies.

## Conclusions

Addressing the root causes of social determinants such as poverty, education, crime, and inequality that promote or deteriorate population health, is integral to improving population health and reducing critical health disparities [58]. This exploratory study highlights the geographic disparities in population health occurring in RI, and supports the preponderance of evidence suggesting that social determinants are associated with population health across the lifespan. Understanding and addressing key upstream drivers of population health and living conditions, especially those that are potentially modifiable through evidence-based policies, programs, and interventions, are critical to promoting health across all demographic groups.

## Data Availability

The data used in this study, the Behavioral Risk Factor Surveillance System, is publicly available on the CDC website (www.cdc.gov/brfss). The datasets used and/or analyzed during the current study are available from the corresponding author on reasonable request.

## Abbreviations

LE0: Life expectancy at birth (age 0)
LE65: Life expectancy at age 65
ASMR: Age-standardized mortality rate (per 100,000)
CDC: Centers for Disease Control and Prevention
RI: Rhode Island
